# Flavonoids from *Morus alba* L. Leaves: Optimization of Extraction by Response Surface Methodology and Comprehensive Evaluation of Their Antioxidant, Antimicrobial, and Inhibition of α-Amylase Activities through Analytical Hierarchy Process

**DOI:** 10.3390/molecules24132398

**Published:** 2019-06-28

**Authors:** Hao Cui, Tenghui Lu, Mingxin Wang, Xintong Zou, Yang Zhang, Xiudong Yang, Yao Dong, Hongli Zhou

**Affiliations:** 1 School of Chemistry and Pharmaceutical Engineering, Jilin Institute of Chemical Technology, Jilin 132022, China; 2 Engineering Research Center for Agricultural Resources and Comprehensive Utilization of Jilin Provence, Jilin Institute of Chemical Technology, Jilin 132022, China; 3 College of Biology & Food Engineering, Jilin Institute of Chemical Technology, Jilin 132022, China

**Keywords:** mulberry leaves, flavonoids, optimization, extraction conditions, in vitro, bioactivities, AHP, evaluate comprehensively

## Abstract

To explore the flavonoids from *Morus alba* L. leaves (MLF), the process of extracting was optimized by a response surface methodology and the antimicrobial and antioxidant activities were evaluated in vitro. The yield of flavonoids reached 50.52 mg g^−1^ under the optimized extraction conditions (i.e., extraction temperature, 70.85 °C; solvent concentration, 39.30%; extraction time, 120.18 min; and liquid/solid ratio, 34.60:1). The total flavonoids were extracted in organic solvents with various polarities, including petroleum ether (MLF_p_), ethyl acetate (MLF_e_), and *n*-butanol (MLF_b_). In vitro, the four MLF samples exhibited good antioxidant activities for scavenging of 2, 2′-azinobis-(3-ethylbenz-thiazoline-6-sulphonate) radical, 1, 1-diphenyl-2-picrylhydrazyl radical, and total reducing power. Regarding antimicrobial efficacy, the MLF samples suppressed the development of *Staphylococcus aureus*, *Bacillus subtilis*, and *Bacillus pumilus*. The MLF samples inhibited α-amylase activity to a certain extent. The analytical hierarchy process (AHP) was used to evaluate comprehensively the bioactivities of the MLF samples. The AHP results revealed that the bioactivity comprehensive score (78.83 μg mL^−1^) of MLF_e_ was optimal among the four MLF samples. *Morus alba* L. leaves also exhibited non-hemolytic properties. All bioactivities suggested the potential of MLF_e_ as a candidate resource in the food and drug industries.

## 1. Introduction

*Morus alba* L. (mulberry) leaves are used in traditional medicine and were even recognized as edible, medicinal substances in 1993 by the Ministry of Health of the People’s Republic of China. Extracts of mulberry leaves contain a series of bioactivity products, including flavonoids, γ-aminobutyric acid, 1-deoxynojirimycin, and chlorophyll [1]. Flavonoids contain active hydrogen, which can terminate the chain reaction of oxygen radicals, scavenge free radicals, and eliminate the toxic effect of radicals [2]. These flavonoids’ antioxidation, anti-hyperlipidemia, anti-fatigue, anti-aging, and atherosclerosis-prevention activities have previously been reported. For example, Li [3] confirmed that flavonoids extracted from mulberry leaves with 60% ethanol solution have anti-fatigue activity in mice. Katsube [4] extracted compounds containing three flavonol glycosides with low density-lipoprotein antioxidant activity and found that quercetin3-(6-malonylglucoside) and rutin are the main flavonol glycosides in mulberry leaves. Kim [5] found that the water-extraction abilities of mulberry leaves containing four flavonols followed the order quercetin-3-β-d-glucose > quercetin-3-*O*-glucose-6-acetate > rutin > quercetin. Compared with its glycosides, quercetin exhibited a stronger cellular antioxidant capacity against 2,2-azobis (2-amidinopropane) dihydrochloride and Cu^2+^-induced oxidative stress in HepG2 cells.

Flavonoids are also well known for their antimicrobial activity. The antimicrobial capacity and antibiotic spectra of flavonoids vary because their components originate from different sources. Liu [6] found seven purified flavonoids from *Halostachys capsica* exhibiting various antimicrobial capacities for eight microorganisms through minimal inhibitory concentration (MIC). Santas [7] found that quercetin and kaempferol inhibit Gram-positive bacteria, such as *Bacillus cereus*, *Staphylococcus aureus*, *Microcroccus luteus*, and *Listeria monocytogenes*. Conversely, Gram-negative ones, such as *Escherichia coli* and *Pseudomonas aeruginosa,* are less sensitive to their antimicrobial effect, and *Candida albicans* is totally resistant.

The flavonoids of luteolin, myricetin, and quercetin are potent inhibitors of α-amylase, whose activity is related to substitution, such as 2,3-double bond, 5-OH, and the linkage of the B ring at the 3-position [8]. Lo [9] revealed the relationship of the structure and activity of α-amylase inhibitors by molecular docking, and results show that the inhibitory activity of flavonoids relies on hydrogen bonds among the hydroxyl groups of polyphenol ligands, the catalytic residues of the binding site, and the formation of a conjugated π-system that stabilizes the interaction with the active site.

The extraction of flavonoids from mulberry leaves is usually carried out through ethanol extraction, ultrasonic extraction, and microwave extraction. Zhang [10] optimized the ethanol extraction of flavonoids from mulberry leaves through a response surface methodology (RSM) and confirmed the optimal process, namely, an ethanol concentration of 71.75%, a temperature of 67.1°C, liquid/solid ratio of 23.2:1, and a time of 150 min. Under these conditions, the flavonoids yield was 2.37%. Wang [11] found a flavonoid yield of 3.50% under the following conditions: ethanol concentration, 70%; liquid/solid ratio, 50:1; time, 90 min; and temperature, 80°C.

The analytical hierarchy process (AHP) is a multi-criterion quantitative analysis technique to solve complex problems of multiple objectives [12]. In this methodology, the issue is resolved in a hierarchy structure, consisting of high, middle, and low levels. The elements in these levels are compared in pairs [13]. Hence, the relative preference is assessed with respect to each element at the next and higher level, based on the subjective judgment of experts. Further mathematical treatment is used to increase the scientific nature.

In the present study, a hot-reflux method was used, and the extraction process was optimized by RSM through single-factor experiment. In vitro activities, including antibacterial, antioxidant, and α-amylase inhibition, of different flavonoids from dried, mature *Morus alba* L. leave samples (MLF) were analyzed. Multiple-bioactivities of MLF samples were evaluated by AHP. The hemolytic property of MLF_e_ was determined to investigate its initial toxicity. Results indicated the potential application of MLF_e_ in the food and drug industries.

## 2. Results

### 2.1. Evaluation of Single Factors Affecting MLF Extraction

**Figure 1:**
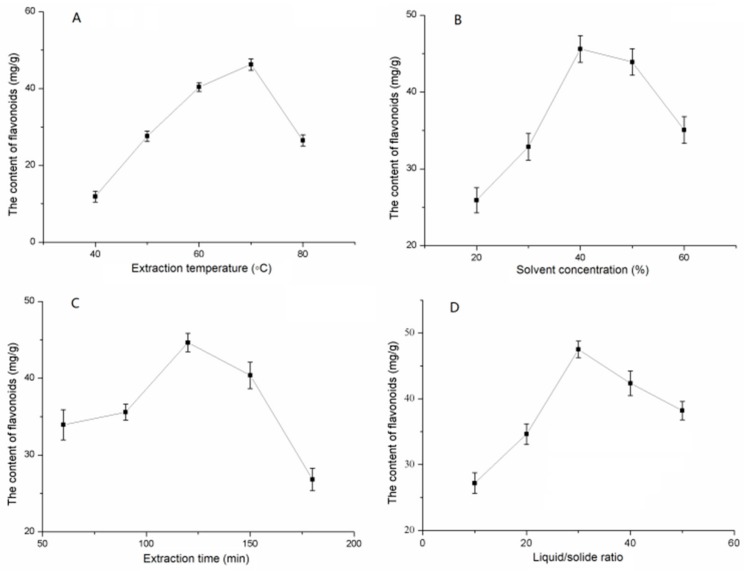
The effect of single factors on the extraction of *Morus alba* L. leaves (MLF). (**A**) Extraction temperature (°C); (**B**) solvent concentration (%); (**C**) extraction time (min); (**D**) liquid/solid ratio. Data are shown as mean ± SD (*n* = 3).

The effects of various temperatures (40–80 °C) on MLF extractions were examined, and the results are presented in Figure 1A. The highest yield was 70 °C; Y_MLF_ increased from 11.87 ± 1.43 mg g^−1^ to 46.25 ± 1.49 mg g^−1^ at 40–70 °C and then decreased to 26.51 ± 1.45 mg g^−1^ at 80 °C. The optimum temperature for flavonoid extraction was deemed to be 70 °C.

Given the high solubility of flavonoids in ethanol, it was used as an extraction solvent. To further research the effect of ethanol concentration on extraction yield, different concentrations from 20% to 60%were tested. Figure 1B shows the effect of solvent on Y_MLF_. The yield of MLF increased from 20% to 40% and reached a peak of 45.60 ± 1.73 mg g^−1^ at 40% concentration, but obviously decreased at 40% to 60%. Accordingly, 40% ethanol was chosen for succeeding experiments.

To investigate the effect of extraction time on Y_MLF_, extraction was carried out at 60–180 min. As shown in Figure 1C, from 60 min to 120 min, the yield prominently increased from 33.94 ± 1.97 to 44.64 ± 1.19 mg g^−1^, and then leveled off. Accordingly, 120 min was used for RSM tests.

The liquid/solid ratio was also investigated. Figure 1D shows the extraction results in different liquid/solid ratios. Extraction efficiency increased with an increased liquid/solid ratio, especially from 27.19 ± 1.57 mg g^−1^ (10:1) to 47.50 ± 1.26 mg g^−1^ (30:1), and then leveled off. Thus, the liquid/solid ratio of 30:1 was selected for subsequent experiments.

### 2.2. RSM Analysis

The extraction of flavonoids was optimized by RSM. Table 1 shows the coded and actual levels used in the optimization process. A total of 29 tests were designed, including 5 zero-point experiments and 24 factorial tests.

Table 2 shows the designed and experimental data of the MLF. The Y_MLF_ ranged within 30.98–50.50 mg g^−1^. By the multivariable regression fitting method of the data in Table 2, the quadratic polynomial regression model of extraction temperature (*A*), solvent concentration (*B*), extraction time (*C*) and liquid/solid ratio (*D*) were generated as shown in the equation blow:

(1) $$R_{1}=49.20+0.31A-0.24B+1.12C+4.81D+2.40AB-4.47AC+2.29AD\;-0.66BC-0.67BD-1.61CD-6.87A^{2}-2.41B^{2}-4.37C^{2}-5.48D^{2}$$

The analysis of variance (ANOVA) for the regression equation is shown in Table 3, with *p* < 0.05, the linear coefficient (*C* and *D*), the quadratic coefficients (*A*^2^, *B*^2^, *C*^2^, and *D*^2^), and the interaction coefficients (*AB*, *AC*, and *AD*) having significant effects. The others, namely, *A*, *B*, *BC*, *BD*, and *CD* with *p* > 0.05, had insignificant effects. Furthermore, the influence of the conditions on Y_MLF_ followed the sequence liquid/solid ratio (*D*), extraction time (*C*), extraction temperature (*A*), and solvent concentration (*B*).

The *p*-value of this model was <0.0001, which indicated that the linear and quadratic terms were highly significant. The *p*-value of the lack of fit was 0.1506 (>0.05), indicating that the experimental data adapted to the model.

**Figure 2:**
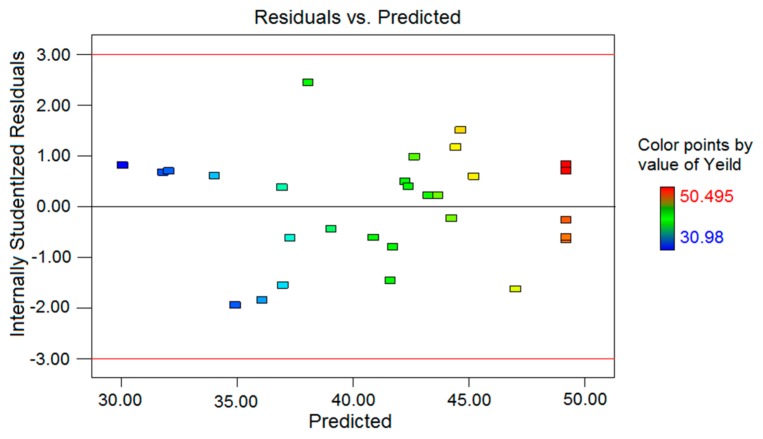
The studentized residuals and predicted response plot.

Figure 2 shows that all residual-standard values were in ±3 intervals, indicating that the model was consistent with the experimental data with no error recorded [14].The precision was 15.292 (>4), indicating an adequate signal and the suitability of this model to be applied in navigation-design space. The “Pred R-Squared” of 0.7620 reasonably agreed with the “Adj R-Squared” of 0.9096. The “Adeq Precision” measures the signal-to-noise ratio, and a ratio greater than four is desirable. The ratio of 15.292 in this work indicated an adequate signal. Thus, this model can be used to navigate the design space.

**Figure 3:**
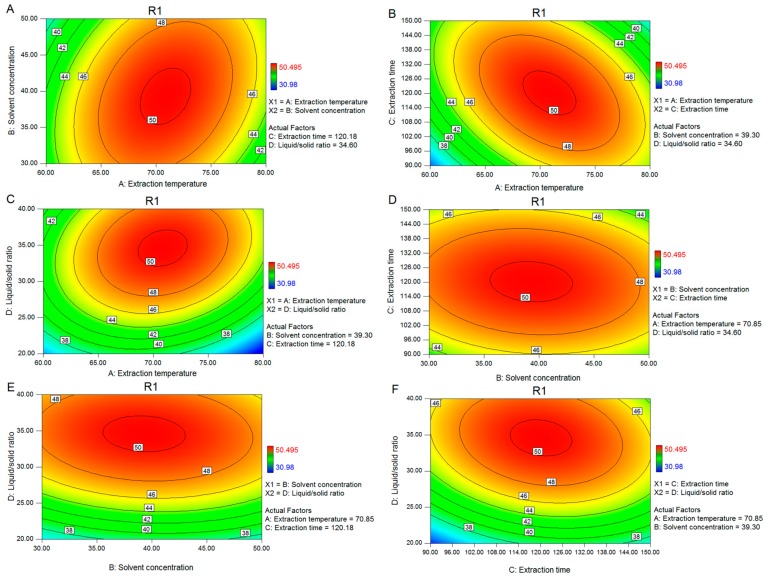
Contour plots showing the effects of the ratio of two single factors. (**A**) Extraction temperature (°C) versus solvent concentration; (**B**) extraction temperature (°C) versus extraction time; (**C**) extraction temperature (°C) versus liquid/solid ratio; (**D**) solvent concentration versus extraction time; (**E**) solvent concentration versus liquid/solid ratio; (**F**) extraction time (min) versus liquid/solid ratio concentration.

Figure 3A shows the contour plot for Y_MLF_ as a function of various extraction temperatures and solvent concentrations at fixed extraction time (120.18 min) and liquid/solid ratio (34.60). The yield of MLF was found to increase rapidly with increased extraction temperature from 60 °C to 70 °C, but decreased rapidly with increased extraction temperature beyond 70 °C. Moreover, Y_MLF_ increased with increased solvent concentration from 30% to 40% and then decreased from 40% to 50%.

Figure 3B shows the contour plot for Y_MLF_ as a function of various extraction temperatures and extraction times at fixed solvent concentration (39.30%) and liquid/solid ratio (34.60). The yield of MLF increased rapidly within the extraction temperature from 60 °C to 70 °C and reached the maximum value. However, after 70 °C, Y_MLF_ did not increase and even decreased to a certain degree. The yield of MLF increased rapidly with the increase of extraction time from 90 min to 120 min, and then decreased slightly from 120 min to 150 min.

Figure 3C shows the contour plot for Y_MLF_ as a function of various extraction temperatures and liquid/solid ratios at a fixed solvent concentration (39.30%) and extraction time (120.18 min). The maximum Y_MLF_ was obtained when the extraction temperature and liquid/solid ratio were 70 °C and 35, respectively.

Figure 3D shows the contour plot for Y_MLF_ as a function of various solvent concentrations and extraction times at a fixed extraction temperature (70.85 °C) and liquid/solid ratio (34.60). The maximum Y_MLF_ was achieved when solvent concentration and extraction time were 40% and 120 min, respectively.

Figure 3E shows the contour plots for Y_MLF_ as a function of various solvent concentrations and liquid/solid ratios at a fixed extraction temperature (70 °C) and extraction time (120 min). The maximum Y_MLF_ was achieved when the solvent concentration and liquid/solid ratio were 40 and 35, respectively.

Figure 3F shows the contour plot for Y_MLF_ as a function of various extraction times and liquid/solid ratios at a fixed extraction temperature (70 °C) and solvent concentration (40%). The yield of MLF increased with prolonged extraction time from 90 min to 120 min and then decreased from 120 min to 150 min. Moreover, Y_MLF_ increased rapidly with increased solvent concentration from 20% to 35% but decreased slightly beyond 35%.

Overall, the optimal conditions for total flavonoids extraction from mulberry leaves were as follows: extraction temperature of 70.85 °C, solvent concentration of 39.30%, extraction time of 120.18 min, and liquid/solid ratio of 34.60:1. The predicted yield was 50.33 mg g^−1^. According to the quadratic polynomial model and the contour plot (Figure 3), the liquid/solid ratio was the most significant factor that influenced Y_MLF_, followed by extraction time, extraction temperature, and solvent concentration.

### 2.3. Verification of Predictive Model

The value predicted using the software was verified under selected optimal extraction conditions. The actual values were examined under the conditions determined from the RSM. The mean of actual values, i.e., 50.52 mg g^−1^, was in accordance with the predicted value indicating that the model was adequate for the extraction process.

### 2.4. The Quantification of Extract of MLF with Different Solvent

The MLF_p_, MLF_e_, and MLF_b_ were generated by extraction of MLF with petroleum ether, ethyl acetate, and *n*-butanol, respectively. The MLF, MLF_p_, MLF_e_, and MLF_b_ were quantified, and the content ratio of the flavonoids was 100:7.33:62:28.67. The total extraction rate of the flavonoids by three solvents was 98%.

### 2.5. Antimicrobial Activity

Three kinds of microorganisms, namely, *S.aureus*, *B.subtilis*, and *B.pumilus* were selected to test the in vitro antibacterial activity of MLF, MLF_p_, MLF_e_, and MLF_b_. The MIC was used to represent antimicrobial activity. As shown in Table 4, the MIC of MLF_e_ was of the lowest among all MLF samples.

### 2.6. In Vitro Antioxidant Activity

The scavenging abilities for the 2,2′-azinobis-(3-ethylbenz-thiazoline-6-sulphonate) (ABTS^+^) radical and 1,1-diphenyl-2-picrylhydrazyl (DPPH) radical, as well as the total reducing power, were selected to investigate the antioxidant activity of MLF in vitro, with vitamin C (VC) as the positive control. The antioxidant activity was characterized with the concentration of MLF samples that resulted in 50% of scavenging (IC_50_) or effective concentration at which 0.5 absorbance units (EC_50_) was observed.

As shown in Table 5, extraction of organic solvent with various polarities significantly affected the antioxidant activities in vitro. The ABTS^+^ and DPPH radical-scavenging abilities and the total reducing power increased with increased concentration of MLF and VC from 20 μg mL^−1^ to 400 μg mL^−1^ and 25 μg mL^−1^ to 125 μg mL^−1^, respectively. The flavonoids extracted with ethyl acetate extracted MLF displayed the lowest IC_50_/EC_50_.

### 2.7. α-Amylase Inhibition Activity

The α-amylase inhibition activity of the MLF samples were tested and controlled with acarbose in the same concentration. Activity was evaluated with the value of IC_50_. As shown in Table 6, the IC_50_ values of MLF, MLF_p_, MLF_e_, MLF_b_, and acarbose were 125.00 ± 10.00, 356.67 ± 16.07, 88.00 ± 3.61, and 67.67 ± 7.51 μg mL^−1^, respectively.

### 2.8. AHP Model for Weight Calculation

**Figure 4:**
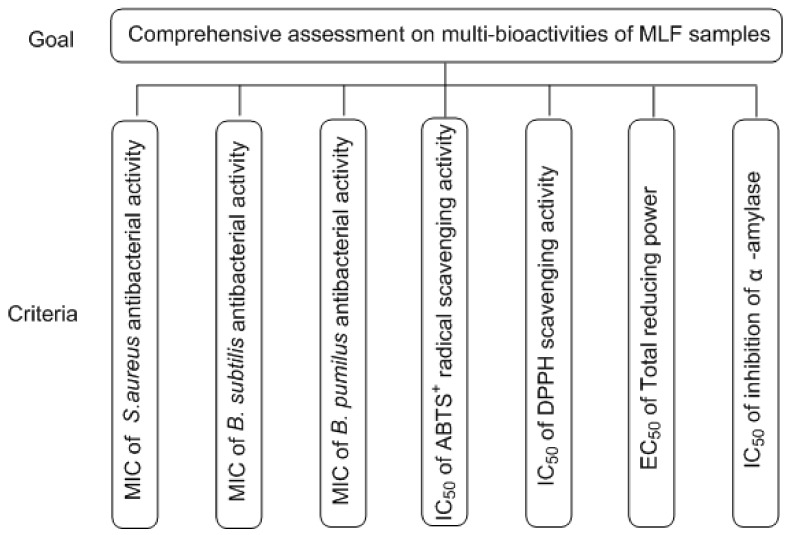
The proposed analytical hierarchy process model.

A multi-criterion model was developed to evaluate the related process parameters, and its structure is depicted in Figure 4. The AHP model consisted of two levels. The top level was the goal of the model (comprehensive assessment of the multiple-bioactivities of MLF samples), and the second level covered the criteria (*f_1_*, MIC of *S.aureus* antibacterial activity; *f_2_*, MIC of *B.subtilis* antibacterial activity; *f_3_*, MIC of *B.pumilus* antibacterial activity; *f_4_*, IC_50_ of ABTS^+^ radical-scavenging activity; *f_5_*, IC_50_ of DPPH scavenging activity; *f_6_*, EC_50_ of total reducing power; and *f_7_*, IC_50_ of α-amylase inhibition activity). The importance of criteria in the goal level was assessed using a suitable scale based on Table 7.

The results were then transformed into positive pairwise comparison matrices N as follows:

(2) $$N=\left[\begin{array}{ccccccc} 1 & 2 & 2 & 3 & 3 & 3 & 5\\ 1/2 & 1 & 1 & 3 & 3 & 3 & 5\\ 1/2 & 1 & 1 & 3 & 3 & 3 & 5\\ 1/3 & 1/3 & 1/3 & 1 & 1 & 1 & 3\\ 1/3 & 1/3 & 1/3 & 1 & 1 & 1 & 3\\ 1/3 & 1/3 & 1/3 & 1 & 1 & 1 & 1/3\\ 1/5 & 1/5 & 1/5 & 1/3 & 1/3 & 3 & 1 \end{array}\right]$$

The calculated initial weights w_1′_, w_2′_, w_3′_, w_4′_, w_5′_, w_6′_, and w_7′_, were 2.4566, 1.8253, 1.8253, 0.7306, 0.7306, 0.5338, and 0.4288, respectively. In further normalized computation, the priority weights w_1_, w_2_, w_3_, w_4_, w_5_, w_6_, and w_7_ were 0.2880, 0.2140, 0.2140, 0.0856, 0.0856, 0.0626, and 0.0503, respectively. The maximum eigenvalue (*λ*_max_) was 7.5182, CI was 0.0864, the consistency ratio (CR) was 0.0654 < 0.1, and the consistency check was passed. The value of w, as the priority weight of criterion to goal, was provided as follows:

(3) $$N=\left[\begin{array}{ccccccc} 0.2880 & 0.2140 & 0.2140 & 0.0856 & 0.0856 & 0.0626 & 0.0503 \end{array}\right]$$

The comprehensive assessment score S of MLF, MLF_p_, MLF_e_, and MLF_b_ were 119.16, 457.45, 78.88, and 169.52, respectively.

### 2.9. Hemolysis Analysis

**Figure 5:**
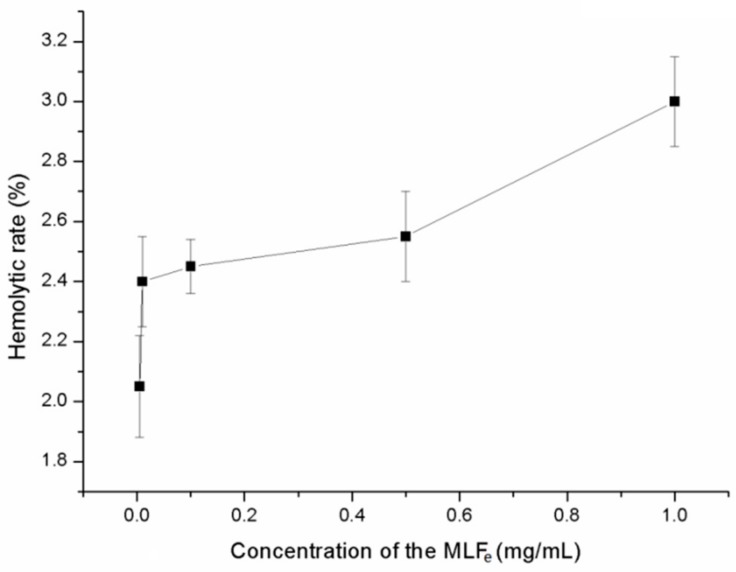
Hemolysis analysis of MLF_e_.

The hemolysis property of MLF was evaluated with the absorbance of human red blood cells (hRBCs), which were treated with the MLF_e_ and quantified in hemolyticrate. As described by Autian [15] and Yang [16], a sample does not have hemolysis property when its hemolyticrate is less than 5%. As shown in Figure 5, MLF_e_ in the concentration range of 0.005–1 mg mL^−1^ had no hemolysis property. Within this range, MLF_e_ exhibited antimicrobial activity, antioxidant activity, and α-amylase inhibition activity. This finding suggested the safe application of MLF_e_ in food and medical industries.

## 3. Discussion

Extraction temperature can surely influence the yield of flavonoids. The increase in temperature can lead to the enhancement of the diffusion rate of solvent and mass transfer, which can improve the dissolution of target compounds. High temperature further promotes the degradation of heat-labile substances. Given the high solubility of flavonoids in ethanol, it was used as an extraction solvent. The extraction time is also an essential factor affecting the extraction yield of compounds [17]. With prolonged extraction time, the yield does not reach its peak value until balance is achieved among the target compounds formed in the extraction system inhabitation and then becomes steady [18]. The liquid/solid ratio is a crucial element as well in the process of extraction. A higher solvent amount corresponds with higher yield, although solvent waste may ensue. In the present work, the extraction temperature, ethanol concentration, extraction time, and liquid/solid ratio were optimized by RSM and found to be 70.85 °C, 39.30%, 120.18 min, and 34.6:1, respectively. The actual yield of MLF was 50.52 mg g^−1^. This result may be due to the multiple impacts of all extraction conditions. No ultimate extraction process existed, as the different combinations of extraction conditions lead to different yields. In Mu’s [19] study, the optimized extraction process by RSM were as follows: an extraction temperature of 68 °C, extraction time of 40 min, ethanol concentration of 52%, and liquid/solid of 86; the yield of mulberry leaf flavonoids was 47.10 mg g^−1^. Huang [20] obtained a mulberry leaf flavonoid yield of 50.2 mg g^−1^ under the following optimized conditions: temperature, (60 °C); solid/liquid ratio, (1:14); ethanol concentration, (70 %); extraction time, (90 min); and number of extractions, 2. In the current study, we obtained the optimized conditions with the minimum ethanol concentration and reasonable liquid/solid ratio, extraction temperature, and extraction time. The yield reached 50.52 mg g^−1^, which was highly suitable for actual production.

The ethanol extract of mulberry leaves contained four flavonoids, and their contents followed the order quercetin 3-(6-malonylglucoside)>rutin >isoquercitrin >astragalin [4]. These flavonoids are well known to have multiple-bioactivities, including antimicrobial, antioxidant, and α-amylase inhibition activity. After treatment with quercetin 3-(6-malonylglucoside), the thiobarbituric acid reactive substances concentrations significantly decreased by 20% in the liver of mice [21]. Rutin exhibited potent activity against *S. aureus* and *B. cereus*, with MIC values of 0.07 and 0.03 mg mL^−1^, respectively [22]. Moreover, the consumption of rutin reduced oxidative stress and glutathione disulfide content, as well as enhanced the levels of glutathione, glutathione peroxidase, glutathione reductase, and glutathione S-transferase in the hepatic tissue of rats with high-fat diet -induced obesity [23]. In vitro, EC_50_ for the DPPH radical scavenging of rutin was 15.7 μg, which approached that of the positive control BHA (9.5 μg) [24]. Isoquercitrin exhibited antioxidant activity in DPPH radical scavenging (IC_50_ = 11.8 μg mL^−1^) [25]. Astragalin possessed antioxidant activities for DPPH radical-scavenging activities (FSC_50_ = 408.00 μmol L^−1^) and reactive oxygen species scavenging activities (OSC_50_ = 8.55 μmol L^−1^). Quercetin, isoquercetin, and rutin can inhibit the activity of α-amylase by binding together in the competitive type [26]. Flavonoids also exhibited good cell-protection effects, for instance, astragalin (1–50 μmol L^−1^) had a half-time 46–90.3 min in hemolysis [27].

The activities of materials with multiple-bioactivities are difficult to evaluate. In the present work, AHP was introduced to calculate the priority weight of bioactivities. As the comprehensive assessment score S is calculated based on the MIC and IC_50_ values, the low S value represented excellent properties. Quercetin 3-(6-malonylglucoside), isoquercitrin, and rutin are highly soluble in ethyl acetate, which explains the antioxidant activity in different plant extracts, including lotus leaf, *Trichosanthis seme*, and *Ipomoea batatas* L. [28,29,30]. Here, the MLF_e_ content of flavonovids in MLF_e_ reached to 62% of the MLF and exhibited multiple-bioactivities and its comprehensive assessment score S was 78.88 μg mL^−1^, which was the highest among all MLF samples. The MLF_e_ at a concentration of 1.0 mg mL^−1^ exhibited no hemolysis property, which indicated the safe application of MLF in the food industry.

## 4. Materials and Methods

### 4.1. Materials and Microbials

Mulberry has been cultivated in Changbaishan for 20 years to accommodate the cold weather. After the first frost, the mulberry leaves were harvested in October 2017 in Jiaohe, Jilin Province and then dried in the shade after washing with distilled water. The dried leaves were pounded in mortars, sifted through a 200 mush sieve, and stored at 4 °C. The rutin standard (code: 100080–201610) was purchased from the National Institute for the Control of Pharmaceutical and Biological Products. All other chemicals and reagents were analytical grade. *Staphylococcus aureus* ATCC 29213, *B.subtilis* ATCC 6633, and *B.pumilus* ATCC 700,814 were purchased from Beijing zhongke quality inspection biotechnology co., LTD (Beijing, China). Healthy hRBCs were obtained from the Red Cross Center for Blood, Jilin, China, which is responsible for the legal administration of human erythrocytes for clinical and scientific usage in Jilin.

### 4.2. Experimental Design and Statistical Analysis

We assumed that the four factors did not influence one another and selected extraction temperature (°C, *A*), solvent concentration (*B*), extraction time (min, *C*), and liquid/solid ratio (*D*) as the signal factors to evaluate their effects on extraction. Seven grams of sample was extracted by hot-reflux method following the conditions in Table 8. The resulting extract was concentrated in rotary evaporators and then in a vacuum drier.

Based on the results of signal-factor experiments, a four-variable, three-level Box-Behnken Design (BBD) was designed with Design-Expert 8.0.6.1 software (Stat-Ease, Inc, Minneapolis, MN, USA) to optimize the extraction. Twenty-nine experimental runs of data were included, and five replicates at the center points were used to evaluate the experimental error and calculate the method repeat ability [31,32]. Second-order polynomial models were developed using the equation as below:

(4) $$Y=\beta_{0}+\overset{n}{\underset{i=1}{\sum }}\beta_{i}X_{i}+\overset{n}{\underset{i=1}{\sum }}\beta_{ii}X_{i}^{2}+\overset{n}{\underset{i<j}{\sum }}\beta_{ij}X_{i}X_{j}+\varepsilon$$

where *Y* is the predicted extraction yield of MLF (Y_MLF_); *β_0_* is the constant coefficient; *β_i_*, *β_ii_*, and *β_ij_* are the first-order, quadratic coefficients of *X_i_*, and effect of interaction, respectively; and *ε* is the experimental error (random). The experimental data were statistically tested by ANOVA through Duncan’s multiple range tests with IBM SPSS, and the results are expressed as the mean ± standard error; *p* < 0.05 indicated significant effects.

After optimization, the adequacy of the model equation was validated by comparing the experimental data with the predicted results of the optimized model.

### 4.3. Determination of Flavonoids Content

The NaNO_2_-Al(NO_3_)_3_-NaOH colorimetric assay described by Xu [33] was used to determine the contents of flavonovids and rutin reference, and the spectrophotometer wavelength was set at 510 nm. The yield (%) was calculated as follows:

(5) $$Y_{\mathrm{MLF}}\left(w/w\right)=\frac{W_{\mathrm{MLF}}\left(\mathrm{mg}\right)}{W_{\mathrm{ML}}\left(g\right)}$$

where Y_MLF_(*w*/*w*), W_MLF_, and W_ML_ are the total flavonoid yield (*w*/*w*), weight of flavonoids (mg), and weight of mulberry leaves (g), respectively.

### 4.4. Extract of MLF with Different Solvent

Solvents such as petroleum ether, ethyl acetate, and n-butanol were used as extract solvents because of their different polarities. Ethanol extracts were extracted from the above solvents, allowing the components in the total flavonoids to be distributed in different solvents according to polarity. The MLF was extracted in organic solvent with various polarities, namely, MLF_p_, MLF_e_, and MLF_b_, based on the method described by Xu [30]. The extracts were concentrated, dried, weighed, quantified, and prepared with different concentrations.

### 4.5. In Vitro Antibacterial Activity Assay

In vitro antibacterial activity was represented with MIC. The micro-dilution method was used in the antibacterial (*S.aureus*, *B. subtilis*, and *B. pumilus*) experiments based on the method described by Cui [34]. Twenty microliters of different concentrations of the samples in 20% DMSO were mixed in a medium made with MLF at a final concentration of 0.50–200.00 μgmL^−1^ in each well. Asolution containing 20% DMSO without MLF was used as a negative control, and 96well plates were placed in a biochemical incubator and cultivated for 24 h.

### 4.6. In Vitro Antioxidant Assay

#### 4.6.1. ABTS^+^ Radical-Scavenging Activity Test

An ABTS^+^ radical-scavenging capacity assay was performed as described by Cui [34]. A 1.0 mL mixture of different concentrations (0.0125–1.35 mg mL^−1^) of the samples and a 1.0 mL of diluted ABTS^+^ radical solution were incubated for 10 min and subjected to absorbance measurement at 734 nm (*A_s_*). The samples were replaced with distilled water in the blank system (*A_0_*), and VC was used as the positive control sample in concentrations of 1–9 μg mL^−1^. The inhibition rate of ABTS^+^ radical scavenging of samples was calculated using the following formula:

(6) $${\mathrm{ABTS}}^{+}\;\mathrm{scavenging}\;\mathrm{activity}\left(\%\right)=\frac{A_{0}-A_{s}}{A_{0}}\times 100\%$$

#### 4.6.2. DPPH Scavenging-Activity Test

The DPPH scavenging-activity test was performed as described by Cui [34]. The MLF samples were dissolved in distilled water to different concentrations (0.05–2.80 mg mL^−1^). Two milliliters of diluted sample solution were mixed with 500 μL of ethanol solution of DPPH (0.2 g L^−1^) and 1.0 mL of ethanol. After allowing standing for 30 min in darkness, the absorbance of the mixture was monitored at 517 nm (*A_s_*). The test system containing DPPH solution without sample and containing ethanol without DPPH solution were used as a control (*A_c_*) and blank (*A_0_*), respectively. Vitamin C was used as a positive control at 1–9 μg mL^−1^. The DPPH radical-scavenging activity was calculated by the following equation:

(7) $$\mathrm{DPPH}\;\mathrm{radical}\;\mathrm{scavenging}\;\mathrm{activity}\left(\%\right)=\left[1-\frac{A_{S}-A_{C}}{A_{0}}\right]\times 100\%$$

#### 4.6.3. Total Reducing Power Test

The total reducing power of the MLF samples was determined according to the method described by Cui [34]. Different flavonoids concentrations (0.2–2.8 mg mL^−1^) in 2 mL of 75% ethanol solution were mixed with PBS (2 mL, 0.2 mol L^−1^, pH 6.6) and potassium ferricyanide (K_3_Fe(CN)_6_) (2 mL, 1%). The mixture was incubated at 50 °C for 20 min. Aliquots (2 mL) of trichloroacetic acid (10%) were added to the mixture. The top layer of the solution (2 mL) was mixed with distilled water (2 mL) and FeCl_3_ (0.5 mL, 0.1%), and the absorbance was measured at 700 nm with a spectrophotometer. Increased absorbance of the reaction mixture indicated an increase in reduction capability. The concentrations of the positive control sample VC was set at 0.05–0.2 μg mL^−1^.

### 4.7. Inhibition of α-Amylase Activity Assay

The α-amylase inhibition-activity assay of MLF samples was conducted based on the method described by Ali [35] with slight modifications. The MLF samples were diluted to different concentrations, i.e., 0.25–10 mg mL^−1^. Twenty microliters of the above sample solution were mixed in 20 μL of α-amylase solution, and then the mixture was incubated at 37 °C for 10 min. A forty microliter starch solution was added into the mixture followed by incubation at 37 °C for 15 min. The reaction was terminated by adding 20 μL of hydrochloric acid. For coloring, 100 μL of I_2_-KI solution was used to treat the mixture, and then the absorbance at 595 nm (*A_s_*) was measured. The two other reaction systems with PBS replacing starch solution and sample were used as the control (*A_c_*) and blank (*A_b_*), respectively. In the positive-control groups, the samples were replaced with acarbose at 9.80–156.25 μg mL^−1^. The α-amylase activity-inhibition rates were calculated by the following formula:

(8) $$\alpha -\mathrm{amylase}\;\mathrm{activityinhibitionrates}\;\left(\%\right)=\frac{A_{s}}{A_{c}-A_{b}}\times 100\%$$

### 4.8. Calculation of Priority Weights by Analytic Hierarchy Process

The priority weights of *S.aureus* antibacterial activity, *B.subtilis* antibacterial activity, *B.pumilus* antibacterial activity, ABTS^+^ radical scavenging-activity, DPPH scavenging-activity, total reducing power, and inhibition of α-amylase were calculated by AHP according to a reported method [36]. The evaluation target tree was initially established, and then a pairwise comparison matrix of the criteria was built. Results of the pairwise comparison were transformed into a positive pairwise comparison matrix N as follows:

(9) $$N={\left[N_{ij}\right]}_{n\times n}=\left[\begin{array}{cccc} f_{1}/f_{1} & f_{1}/f_{2} & \cdots & f_{1}/f_{n}\\ f_{2}/f_{1} & f_{2}/f_{2} & \cdots & f_{2}/f_{n}\\ \vdots & \vdots & \vdots & \vdots \\ f_{n}/f_{1} & f_{n}/f_{2} & \cdots & f_{n}/f_{n} \end{array}\right]i,j=1,2\cdots \;n$$

where *f_i_*/*f_j_* represents the priority weights of factors *i* and *j*. The weights were scored in nine possible scales, as shown in Table7.

The initial and normalized weight coefficient, w_i_’ and w_i_, were calculated as follows:

(10) $$w_{i}^{\prime }=\sqrt[{n}]{f_{1}f_{2}\cdots f_{n}}\;w_{i}=\frac{w_{i}^{\prime }}{\sum_{i=1}^{n}w_{i}^{\prime }}$$

The consistency ratio (CR) of the pairwise matrix was used to evaluate the reasonability and calculated as follows:

(11) $$\mathrm{CR}=\frac{\mathrm{CI}}{\mathrm{RI}}\;\mathrm{CI}=\frac{\lambda_{\max}-n}{n-1}\;\lambda_{\max}=1/n\sum_{i=1}^{n}(\sum_{j=1}^{n}f_{ij}\times \frac{w_{j}}{w_{i}})$$

where RI is the random consistency index whose value for computation is presented in Table 9 [37].

If CR ≤ 0.1, the evaluation within the matrix was acceptable. The normalized weight coefficient was then used to comprehensively assess the multiple-bioactivities of the MLF samples; if CR > 0.1, the judgment was “untrustworthy”. The comprehensive-assessment score of the multiple-bioactivities was measured as S:

(12) $$S=\sum_{i=1}^{n}f_{i}\times \mathrm{wi}$$

### 4.9. The Hemolytic Examination of Flavonoids

The hemolytic test was performed per the description of the Chinese Pharmacopoeia. Thirty microliters of different concentrations of MLF_e_ were added into amixture of 250 μL of 2% hRBC suspension, and 220 μL of 0.9% NaCl solution was added to the MLF_e_ until the final concentrations of 5, 10, 100, 500, and 1000 mg mL^−1^ were reached. The system was incubated at 37 °C for 1 h and centrifuged at 1000 r min^−1^ for 10 min. One hundred microliters of supernatant were transferred onto a 96well plate, and the absorbance was measured at 540 nm (*A_MLF__e_*) with Molecular Devices, LLC Spectra Max ^®^ M4 (Sunnyvale, CA, USA). The system without MLF_e_, which was replaced with the same volume 0.9% NaCl solution, was set as the negative control (*A_N_*). The system contained 250 μL of 2% hRBC suspension, and 250 μL of distilled water was set as the positive control (*A_P_*). Amixture of 470 μL of 0.9% NaCl solution and 30 μL of MLF_e_ sample was set as the background (*A_B_*). The tests were run in triplicate. Data were expressed as the mean ± standard deviation. The hemolyticrate was calculated with the following formula:

(13) $$\mathrm{Hemolytic}\;\mathrm{rate}=\frac{A_{MLFe}-A_{N}-A_{B}}{A_{P}-A_{N}}$$

### 4.10. Statistical Analysis

All assays were performed in triplicate. The experimental data were statistically tested by ANOVA through Duncan’s multiple-range tests by using SPSS statistical software version 19.0 (SPSS Inc, Chicago, IL, USA). Results are expressed as the mean ± standard error; *p* < 0.05 indicated significant effects.

## 5. Conclusions

The extraction conditions of MLF were optimized by BBD and found to be as follows: extraction temperature of 70.85 °C, solvent concentration of 39.30%, extraction time of 120.18 min, and liquid/solid ratio of 34.60:1. The yield under these optimized conditions was 50.52 mg g^−1^. The good antimicrobial, antioxidant, and α-amylase inhibition activity of MLF_e_ suggested its potential usage in the food industry, such as a meat preservative, or in the drug industry, such as antibacterial and hypoglycemic agents. No hemolysis property was observed at 1 mg mL^−1^, which was higher than the working concentration, thereby indicating the safety application of MLF_e_.

## Figures and Tables

**Table 1:** Coded and actual levels of independent variables used in the RSM.

Independent Variables	Symbol	Coded Levels		
		−1	0	+1
Extraction temperature (°C)	A	60 °C	70 °C	80 °C
Solvent concentration	B	30%	40%	50%
Extraction time (min)	C	90	120	150
Liquid/solid ratio	D	20:1	30:1	40:1

**Table 2:** Response surface design and experimental data.

Run	Coded Solvent Concentration Variable	Coded Liquid/Solid Ratio Variable	Coded Time Variable	Coded Temperature Variable	Predicted Value (mg g^−1^)	Actual Value (mg g^−1^)
1	0	+1	−1	0	41.73	40.84
2	0	−1	0	−1	36.08	34.01
3	+1	−1	0	0	38.07	40.81
4	+1	0	0	−1	30.06	30.98
5	−1	0	0	−1	34.03	34.71
6	0	0	0	0	49.20	48.17
7	−1	0	−1	0	32.06	32.84
8	0	0	−1	+1	44.65	46.35
9	+1	0	0	+1	44.26	44.00
10	0	0	+1	+1	43.67	43.91
11	−1	0	0	+1	39.06	38.56
12	0	0	0	0	49.20	48.26
13	−1	+1	0	0	36.98	35.24
14	0	+1	+1	0	42.66	43.76
15	0	+1	0	+1	45.22	45.89
16	0	−1	−1	0	40.88	40.19
17	−1	0	+1	0	43.24	43.49
18	0	−1	+1	0	44.44	45.75
19	0	0	0	0	49.20	48.79
20	0	−1	0	+1	47.03	45.20
21	+1	0	+1	0	34.92	32.73
22	+1	0	−1	0	41.62	39.97
23	0	0	−1	−1	31.81	32.57
24	0	+1	0	−1	36.95	37.37
25	+1	+1	0	0	42.40	42.85
26	0	0	0	0	49.20	50.50
27	−1	−1	0	0	42.26	42.81
28	0	0	+1	−1	37.28	36.59
29	0	0	0	0	49.20	50.30

**Table 3:** Variance analysis of extracted equation of flavonoids from mulberry leaves.

Source	Sum of Squares	df	Mean Square	F Value	*p*-Value Prob > F	
Model	895.77	14	63.98	21.13	<0.0001	significant
*A*-Extraction temperature	1.14	1	1.14	0.38	0.5492	
*B*-Solvent concentration	0.66	1	0.66	0.22	0.6471	
*C*-Extraction time	15.12	1	15.12	4.99	0.0423	
*D*-Liquid/solid ratio	277.25	1	277.25	91.55	<0.0001	
*AB*	23.06	1	23.06	7.62	0.0154	
*AC*	79.88	1	79.88	26.38	0.0002	
*AD*	21.02	1	21.02	6.94	0.0196	
*BC*	1.74	1	1.74	0.57	0.4616	
*BD*	1.78	1	1.78	0.59	0.4557	
*CD*	10.40	1	10.40	3.43	0.0850	
*A* ^2^	306.19	1	306.19	101.10	<0.0001	
*B* ^2^	37.53	1	37.53	12.39	0.0034	
*C* ^2^	123.97	1	123.97	40.94	<0.0001	
*D* ^2^	194.65	1	194.65	64.27	<0.0001	
Residual	42.40	14	3.03			
Lack of Fit	37.41	10	3.74	3.00	0.1506	not significant
Pure Error	4.99	4	1.25			
Cor Total	938.17	28				

**Table 4:** MIC of antibacterial activity results of MLF.

	*Staphylococcus aureus*	*Bacillus subtilis*	*Bacillus pumilus*
MLF (μg mL^−1^)	30.24	30.24	18.14
MLF_p_ (μg mL^−1^)	50.40	140.00	50.40
MLF_e_ (μg mL^−1^)	3.92	10.89	6.53
MLF_b_ (μg mL^−1^)	50.40	84.00	30.24

**Table 5:** The results of antioxidant in vitro of MLF samples and vitamin C (VC).

	ABTS^+^ (IC_50_)	DPPH (IC_50_)	Reducing Power (EC_50_)
MLF (μg mL^−1^)	60.33 ± 5.51 ^a^	211.67 ± 7.64 ^a^	1126.67 ± 64.29 ^a^
MLF_p_ (μg mL^−1^)	642.33 ± 11.24 ^b^	2070.00 ± 60.83 ^b^	2429.33 ± 112.88 ^b^
MLF_e_ (μg mL^−1^)	32.73 ± 1.07 ^c^	145.00 ± 13.23 ^c^	868.67 ± 18.04 ^c^
MLF_b_ (μg mL^−1^)	44.33 ± 6.03 ^ac^	154.00 ± 9.64 ^c^	1760.00 ± 52.92 ^d^
VC (μg mL^−1^)	7.20 ± 0.70 ^d^	0.0047 ± 0.00055 ^d^	0.079 ± 0.0032 ^e^

^a,b,c,d,e^ Same antioxidant system with different superscripts are significantly different (*p* < 0.05).

**Table 6:** α-Amylase inhabitation of the MLF.

	α-Amylase Inhabitation Activity (IC_50_)
MLF (μg mL^−1^)	125.00 ± 10.00 ^a^
MLF_p_ (μg mL^−1^)	356.67 ± 16.07 ^b^
MLF_e_ (μg mL^−1^)	88.00 ± 3.61 ^c^
MLF_b_ (μg mL^−1^)	66.67 ± 7.05 ^d^
Acarbose (μg mL^−1^)	51.33 ± 4.04 ^e^

^a,b,c,d,e^ Different superscripts are significantly different (*p* < 0.05).

**Table 7:** Value meaning of the nine-scale method.

Description of *f_i_*/*f_j_*	Scale
*f_i_* is equally important as *f_j_*	1
*f_i_* is moderately more important than *f_j_*	3
*f_i_* is strongly more important than *f_j_*	5
*f_i_* is very strongly more important than *f_j_*	7
*f_i_* is extremely more important than *f_j_*	9
The median of adjacent scales, when a compromise is needed	2, 4, 6, 8

**Table 8:** The extraction conditions of single element test.

	Ethanol Concentration	Liquid/Solid Ratio	Extraction Temperature	Extraction Time
Ethanol concentration	20%, 30%, 40%, 50%, 60%	30: 1	70 °C	90 min
Liquid/solid ratio	70%	10:1, 20:1, 30:1, 40:1, 50:1	60 °C	90 min
Extraction temperature	70%	30:1	40, 50, 60, 70, 80 °C	90 min
Extraction time	50%	20:1	70 °C	60, 90, 120, 150, 180 min

**Table 9:** The average consistencies of random matrices.

Matrix order	1	2	3	4	5	6	7	8	9
Random consistency index	0.00	0.00	0.58	0.90	1.12	1.24	1.32	1.41	1.45

## References

[1] Vichasilp C., Nakagawa K., Sookwong P., Suzuki Y., Kimura F., Higuchi O., Miyazawa T. (2009). Optimization of 1-deoxynojirimycin extraction from mulberry leaves by using response surface methodology. Biosci. Biotechnol. Biochem..

[2] Han R.M., Zhang J.P., Skibsted L.H. (2012). Reaction dynamics of flavonoids and carotenoids as antioxidants. Molecules.

[3] Li W., Li T., Tang K. (2009). Flavonoids from mulberry leaves by microwave-assisted extract and anti-fatigue activity. Nurs. Clin. North Am..

[4] Katsube T., Imawaka N., Kawano Y., Yamazaki Y., Shiwaku K., Yamane Y. (2006). Antioxidant flavonol glycosides in mulberry (*Morus alba* L.) leaves isolated based on LDL antioxidant activity. Food Chem..

[5] Kim G.N., Jang H.D. (2011). Flavonol content in the water extract of the mulberry (*Morus alba* L.) leaf and their antioxidant capacities. J. Food Sci..

[6] Liu H., Mou Y., Zhao J., Wang J., Zhou L., Wang M., Wang D., Han J., Yu Z., Yang F. (2010). Flavonoids from *Halostachys caspica* and their antimicrobial and antioxidant activities. Molecules.

[7] Santas J., Almajano M.P., Carbó R. (2010). Antimicrobial and antioxidant activity of crude onion (*Allium cepa*, L.) extracts. Int. J. Food Sci. Technol..

[8] Tadera K., Minami Y., Takamatsu K., Matsuoka T. (2007). Inhibition of α-glucosidase and α-amylase by flavonoids. J. Nutr. Sci. Vitaminol..

[9] Lo P.E., Scheib H., Frei N., Williamson G., Grigorov M., Chou C.J. (2008). Flavonoids for controlling starch digestion: Structural requirements for inhibiting human alpha-amylase. J. Med. Chem..

[10] Zhang H.W., Chen X.Y., Ling C.Y. (2010). Optimization of alcoholic extraction of flavonoids from mulberry leaves using response surface methodology. Amino Acids Biotic Resour..

[11] Wang F., Qiao L., Dan X.Y., Chu F.J. (2011). Study on extraction and anti-oxidation of flavonoids from *Morus alba* L. leaves. Guangdong Agric. Sci..

[12] Saaty T.L. (1980). The Analytic Hierarchy Process.

[13] Herva M., Roca E. (2013). Review of combined approaches and multi-criteria analysis for corporate environmental evaluation. J. Clean. Prod..

[14] Hasni K., Ilham Z., Dharma S., Varman M. (2017). Optimization of biodiesel production from *Brucea javanica*, seeds oil as novel non-edible feedstock using response surface methodology. Energy Convers. Manage..

[15] Autian J. (1975). Biological model systems for the testing of the toxicity of biomaterials. Polym. Med. Surg..

[16] Yang X.C., Niu Y.L., Zhao N.N., Mao C., Xu F.J. (2014). A biocleavable pullulan-based vector via atrp for liver cell-targeting gene delivery. Biomaterials.

[17] Galhiane M.S., Rissato S.R., Chierice G.O., Almeida M.V., Silva L.C. (2006). Influence of different extraction methods on the yield and linalool content of the extracts of *Eugenia uniflora* L.. Talanta.

[18] Dong J., Liu Y., Liang Z., Wang W. (2010). Investigation on ultrasound-assisted extraction of salvianolic acid B from *Salvia miltiorrhiza* root. Ultrason. Sonochem..

[19] Mu H.R., Chen K., Wang X.L., Liu Y.F., Xin X.D. (2016). Comparison of orthogonal design and response surface methodology used to optimization of flavonoids extraction from mulberry leaves. J. Jiangsu Univ. Sci. Technol..

[20] Huang Q. (2016). Optimization of Extraction Process of Flavonoids from Mulberry Leaves. Food Res. Dev..

[21] Takuya K., Masayuki Y., Kuninori S., Tomoko I., Ichiro M., Keiko A., Yukikazu Y. (2010). Effect of flavonol glycoside in mulberry (*Morus alba* L.) leaf on glucose metabolism and oxidative stress in liver in diet-induced obese mice. J. Sci. Food Agric..

[22] Singh M., Govindarajan R., Rawat A.K.S., Khare P.B. (2008). Antimicrobial flavonoid rutin from *Pteris vittata* L. against pathogenic *Gastrointestinal microflora*. Am. Fern J..

[23] Chinlin H., Wu C.H., Huang S.L., Gowchin Y. (2009). Phenolic compounds rutin and o-coumaric acid ameliorate obesity induced by high-fat diet in rats. J. Agric. Food. Chem..

[24] Kim S.Y., Gao J.J., Lee W.C., Ryu K.S., Lee K.R., Kim Y.C. (1999). Antioxidative flavonoids from the leaves of *Morus alba*. Arch. Pharm. Res..

[25] Silva C.G., Raulino R.J., Cerqueira D.M., Mannarino S.C., Pereira M.D., Panek A.D., Silva J.F.M., Menezes F.S., Eleutherio E.C.A. (2009). In vitro and in vivo determination of antioxidant activity and mode of action of isoquercitrin and *Hyptis fasciculata*. Phytomedicine.

[26] Li Y., Gao F., Gao F., Shan F., Bian J., Zhao C. (2010). Study on the interaction between 3 flavonoid compounds and alpha-amylase by fluorescence spectroscopy and enzymatic kinetics. J. Food Sci..

[27] Park S.N., Sun Y.K., Lim G.N., Na R.J., Min H.L. (2012). In vitro, skin permeation and cellular protective effects of flavonoids isolated from *Suaeda asparagoides* extracts. J. Ind. Eng. Chem..

[28] Zhang L., Wang H.H., Yan S.H., Tu Z.C., Li J., Chen J., Huang Y.Z. (2019). Characterization of the chemical constituents in the ethyl acetate fraction of *Lotus* leaf by UPLC-QTOF-MS/MS. Food Sci..

[29] Liang G.P., Yang J., Liu L., Chen R.H., Ding L.N., Wan L.P., Chen D.L. (2019). Study on α-glucosidase inhibitory acticity and composition analysis of ethylcetate extract from introduced *Trichosanthis Semen* in Guizhou province. West. Chin. J. Pharm. Sci..

[30] Xu H., Zhang Z., Gao Y., Zhang J., Dong Y., Cui H. (2018). Determination of phenolic composition of active extracts from sweet potato leaves and their application in the preservation of chilled pork. Mod. Food Sci. Technol..

[31] Liu Z., Mei L., Wang Q., Shao Y., Tao Y. (2014). Optimization of subcritical fluid extraction of seed oil from *Nitraria tangutorum*, using response surface methodology. LWT Food Sci. Technol..

[32] Nyam K.L., Tan C.P., Lai O.M., Long K., Man Y.B.C. (2010). Optimization of supercritical fluid extraction of phytosterol from roselle seeds with a central composite design model. Food Bioprod. Process..

[33] Xu G. (2007). Studies on the extracting and antioxidant activities of flavonoids in sweet potatoes. J. Food Sci. Biotechnol..

[34] Cui H., Pan H.W., Wang P.H., Yang X.D., Zhai W.C., Dong Y., Zhou H.L. (2018). Essential oils from *Carex meyeriana Kunth*: Optimization of hydrodistillation extraction by response surface methodology and evaluation of its antioxidant and antimicrobial activities. Ind. Crops Prod..

[35] Ali H., Houghton P.J., Soumyanath A. (2006). Alpha-amylase inhibitory activity of some malaysian plants used to treat diabetes; with particular reference to *Phyllanthus amarus*. J. Ethnopharmacol..

[36] Fabjanowicz M., Bystrzanowska M., Namieśnik J., Tobiszewski M., Płotka-Wasylka J. (2018). An analytical hierarchy process for selection of the optimal procedure for resveratrol determination in wine samples. Microchem. J..

[37] Jensen R.E. (1984). An alternative scaling method for priorities in hierarchical structures. J. Math. Psychol..

